# Press-Coated Aceclofenac Tablets for Pulsatile Drug Delivery: Formulation and In Vitro Evaluations

**DOI:** 10.3390/ph15030326

**Published:** 2022-03-08

**Authors:** Rizwana Rashid, Muhammad Zaman, Mahmood Ahmad, Mahtab Ahmad Khan, Muhammad Hammad Butt, Ahmad Salawi, Yosif Almoshari, Meshal Alshamrani, Rai Muhammad Sarfraz

**Affiliations:** 1 Faculty of Pharmacy, University of Central Punjab (UCP), Lahore 54000, Pakistan; rizwanarashid242@gmail.com (R.R.); dr.mahmood@ucp.edu.pk (M.A.); mahtab.ahmad@ucp.edu.pk (M.A.K.); hmdbut@ucp.edu.pk (M.H.B.); 2 Department of Pharmaceutics, College of Pharmacy, Jazan University, Jazan 45142, Saudi Arabia; asalawi@jazanu.edu.sa (A.S.); yalmoshari@jazanu.edu.sa (Y.A.); malshamrani@jazanu.edu.sa (M.A.); 3 College of Pharmacy, University of Sargodha, Sargodha 40100, Pakistan; sarfrazrai85@yahoo.com

**Keywords:** HPMC, HEC, NSAIDs, FTIR, pulsatile drug delivery

## Abstract

The symptoms of some diseases show circadian rhythms, such as the morning stiffness associated with pain at the time of awakening in rheumatoid arthritis. Therapy for such diseases doesn’t require immediate release or sustained release of medicament. In such therapies, pulsatile drug release is more suitable with a programmed drug release. The purpose of this research was to formulate press-coated aceclofenac tablets for pulsatile drug delivery with a distinct delay time of no drug release and release of the drug when it is more likely desired (i.e., after 5 to 6 h). Immediate release core tablets having aceclofenac were formulated. Three formulations, F1, F2, and F3, were prepared with variable concentrations of sodium croscarmellose. Pre- and post-compression tests were performed on the core tablets. The selection criteria included the lowest disintegration time as a requirement of pulsatile drug delivery with an immediate release core and a delayed release coat. The disintegration times of F1, F2, and F3 were 120 s, 60 s, and 15 s, respectively. Therefore, the F3 formulation was selected as the core tablet formulation because it had the shortest disintegration time (15 s). The core tablets were press-coated using different polymers, such as HPMC K100M, Eudragit L100, HEC, and HPMC E5. The polymers were used in the coatings to hinder the release of the core for the desired time. 36 formulations of polymer were prepared: A1 to A10 had HPMC K100M and Avicel PH102; formulations B1 to B6 had HPMC K100M, Eudragit L100, and Avicel PH102; formulations C1 to C7 had HPMC K100M and hydroxyethyl cellulose; formulations D1 to D7 had HPMC K100M and HPMC E5; and formulations E1 to E6 had changed the coating weight of the formulation used for D6 (having HPMC K100M and HPMC E5 in the ratio of 12.5% to 87.5%). Evaluations of the press-coated tablets were carried out through thickness, hardness, weight variation, friability, and in vitro dissolution tests. These parameters concluded that the formulation of E6, having HPMC K100M and HPMC E5 in the ratio of 12.5% to 87.5% at 600 mg weight, was the most optimum formulation as it showed 3.5% drug release after 4 h, 21.4% drug release after 5 h, and 99.27% drug release after 6 h.

## 1. Introduction

For specific treatments, a pulsatile drug release design displays significant advantages, where the active drug releases after a very much characterized lag time. Many body functions show circadian rhythms, for example, gastric pH, cardiac rate, blood flow, blood pressure, stroke volume, and body temperature. Further, the functions of various organs fluctuate with time. It is progressively perceived that in the appearance of numerous illness conditions, there exists a rhythmic and temporal pattern. The indications for various diseases, for example, rheumatic disease, asthma, hypertension, angina pectoris, and myocardial infarction, show circadian rhythms [1]. Circadian rhythms are endogenous oscillations and self-supporting systems which happen with a periodicity of around twenty-four hours. Typically, circadian rhythms are synchronized by internal natural clocks identified with the sleep–awake cycle. As numerous disease states show circadian patterns, benefits could be gained by adjusting the timing of active drug release and, likewise, the administration of medications. The formulation of a drug in such a dosage system that is administered at sleep time, with a personalized start of active drug release in the beginning hours of the morning, can provide a more beneficial treatment compared to the average sustained-release delivery system, given that more reasonable drugs are administered [2]. A pulsatile discharge framework is described as a quick and complete active drug discharge after a lag time. The majority of pulsatile frameworks are reservoir frameworks, typically surrounded by a barrier. After a predetermined time-frame, this outer barrier can be removed, dissolved, or eroded, after which the medication is dissolved and quickly released [1,2,3]. Pain, along with morning stiffness at awakening, is a diagnostic characteristic of rheumatoid arthritis, and such conditions of clinical circadian manifestations are probably the result of the abnormal working of the hypothalamus. Chrono-pharmacotherapy for rheumatoid joint inflammation is needed to ensure that the highest levels of the medication harmonize with the maximum stiffness and pain associated with the disease. The presently available aceclofenac tablets consist of immediate release or extended release dosage forms. A pulse release drug delivery framework that can be given at bedtime, yet delivers the active drug starting in the early hours of the morning, will be an ideal chrono-pharmaceutical framework.

Aceclofenac belongs to the class of nonsteroidal anti-inflammatory drugs (NSAIDs), with well-recognized anti-inflammatory and analgesic characteristics used to treat osteoarthritis, rheumatoid arthritis, and ankylosing spondylitis. It strongly inhibits the cyclo-oxygenase enzyme (COX) which is responsible for the synthesis of prostaglandins, which are the inflammatory mediators that cause pain, swelling, inflammation, and fever. It shows a high permeability to penetrate into synovial joints, where in patients with osteoarthritis and related conditions, the loss of articular cartilage in the area causes joint pain, tenderness, stiffness, crepitus, and local inflammation [4,5]. The anti-inflammatory and analgesic properties of aceclofenac are similar to other NSAIDS; however, preclinical studies show that aceclofenac has less ulcerogenic potential than diclofenac [6].

The current project focused on the development and characterization of press-coated, pulsatile release aceclofenac tablets. The press-coated tablet formulation was optimized by using different concentrations of polymers to obtain a formulation with a definite lag phase of no drug discharge, followed by fast drug discharge after a lag time of about five to six hours, with further polymer effect on drug release and lag time. The press-coated tablets consisted of a quick release core tablet (having aceclofenac) that was press-coated with different proportions of polymers of various viscosity grades (e.g., HPMC E5, HPMC K100M, and hydroxyethyl cellulose).

## 2. Results and Discussion

### 2.1. Pre-Formulation Studies

In Table 1, the results of the pre-formulation studies of aceclofenac are mentioned, including physical appearance, melting point, loss in drying, and drug assay. The values were found to be within the specified limits mentioned in the British Pharmacopoeia [7]. The drug compatibility with excipients was tested by performing FTIR. The resulting spectrum showed that aceclofenac is compatible with all the excipients used in the formulations. The spectrum of FTIR of the formulation showed all the bands that were visible in the spectrum of the pure drug aceclofenac, which confirmed that the drug was compatible with the other components of the formulation, and it was not degraded by the process of forming the tablets. This was also indicative of the aspect that no chemical reaction had occurred between aceclofenac and the other excipients, which was as desired. 

The pre-formulation studies included a physical analysis of the drug by observing its color and texture. Aceclofenac appeared to be a white crystalline powder with a smooth texture. The BP specifications for loss on drying suggest that the value showed should not exceed 0.5, and the value for aceclofenac came out to be 0.2%, which complied with the standards established by BP [8]. 

### 2.2. Pre-Compression Parameters

In Table 2, the results of the pre-compression parameters of the powder blend were checked, such as bulk density, tapped density, angle of repose, Carr’s index, and Hausner’s ratio.

### 2.3. Bulk Density

The bulk density of all formulations was in the range of 0.55 gm/cm^3^ to 0.56 gm/cm^3^. The results depict that good powder flow was present in the formulated tablets, and similar results were reported in the literature [9], which described that the use of bulk density measurements was used as a flowability indicator.

### 2.4. Tapped Density

The tapped densities of all formulations were in the range of 0.60 gm/cm^3^ to 0.62 gm/cm^3^, which was found to be within the acceptable criteria, as mentioned in the methodology.

### 2.5. Angle of Repose

The angle of repose of all formulations was in the range of 27° to 28°. All preparations prepared by the direct compression method showed an angle of repose of less than 30°, which reveals a good flow property according to Geldart and his colleagues’ work on “Characterization of powder flowability using measurement of angle of repose” [10].

### 2.6. Carr’s Index

The Carr’s index for all formulations was in the range of 8.19% to 9.67%, which shows good flow and compressibility properties. According to Geldart et al., powders having a Carr’s index of less than 15% show good flow and compressibility properties, and those with a value of higher than 25% show poor flow and compressibility properties [10].

### 2.7. Hausner’s Ratio

The Hausner’s ratio of all formulations was found to be in the range of 1.09 to 1.11, which shows an excellent flow rate for all formulations. According to Geldart et al., powders having Hausner’s ratios of between 1–1.11 show an excellent flow rate, whereas powders having Hausner’s ratios of greater than 1.60 show a very poor flow rate. The Hausner’s ratio is used as a parameter to check the flow properties of granules and powders in the pharmaceutical industries [10].

### 2.8. Post-Compression Parameters

In Table 3, the results of the post-compression core tablets, such as diameter, thickness, friability, content uniformity, etc., are tabulated. In Table 4, the results of the press-coated tablets are tabulated.

### 2.9. Diameter

All the core tablets were compressed on a 9.0 mm round punch. The diameters of all the core formulations were within the range of 9.001 mm to 9.01 mm. The results showed that all the formulations had a uniform diameter.

### 2.10. Thickness

The thickness of all the core tablets was within the range of 2.91 mm to 2.96 mm. The thickness acceptance criteria were 2.93 mm ± 0.5 mm.

### 2.11. Friability

The friability of F1 was found to be 0.5%, while the friability of F2 and F3 was found to be 0.2%. The results were found to be within the official limit, which is not more than 1%. 

### 2.12. Average Weight

The average weight of all the core tablet formulations and press-coated tablets was found to be within the specified limit mentioned in British Pharmacopoeia, 2016 [11].

### 2.13. Hardness

The hardness of the core tablets was in the range of 5.30 kp to 5.56 kp. The hardness acceptance criterium is not less than 4.0 kp, or 40 N, as this is the hardness required of tablets to withstand the pressure of being press-coated for further processing [12].

### 2.14. Assay and Content Uniformity

The assay of formulations F1, F2, and F3 was 99.92%, 99.94%, and 99.98%, respectively. The uniformity of the content of F1, F2, and F3 was 99.87%, 101.25%, and 99.97%, respectively. The acceptance criteria for the uniformity of the assay and the content are 90% to 110% and 85% to 115%, respectively.

### 2.15. Disintegration Time

The disintegration times of F1, F2, and F3 were 120 s, 60 s, and 15 s, respectively. The reason for this variation in disintegration time is due to percentage of disintegrant used in F1, which was 0.5 percent, while in F3 it was 2.5% and in F2 it was 1%. The official limit for disintegration of the core tablets is not more than 15 min. Khalil and coworkers prepared pulsatile drug release tablets by using different concentrations of super-disintegrants, as for pulsatile delivery, an immediate-release core tablet is required, the outside of which is polymer-coated [13]. Thus, from the results, the formulation of F3 was selected as it had the lowest disintegration time.

### 2.16. FTIR Studies

**Figure 1:**
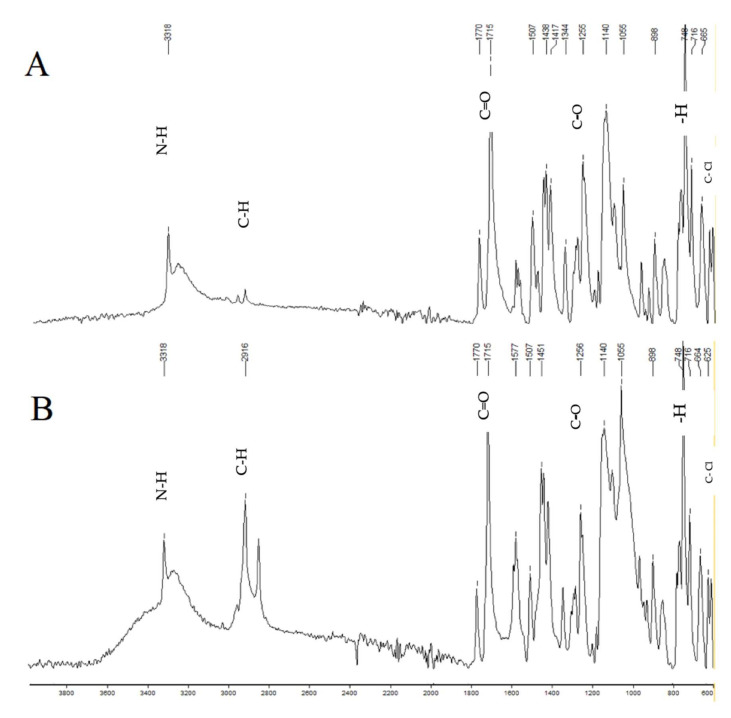
FTIR of aceclofenac (**A**) and press-coated tablet with HPMC E5 (**B**). The characteristic peaks of the aceclofenac spectrum are 1715, which shows C=O stretching; 1255, which shows C-O stretching; 3318, which shows N-H stretching; 2937, which shows C-H stretching; 748, which shows aromatic—H stretching; and 610, which shows C-Cl stretching.

FTIR studies were performed to observe drug–excipient interactions for the physical mixtures of drug with each excipient. It was clearly shown from the results that all characteristic peaks of aceclofenac were present in the combination spectrum, demonstrating the compatibility of the drug and the other excipients used in the formulation of the pulsatile drug delivery system (Figure 1).

### 2.17. In Vitro Dissolution Studies

In vitro dissolution studies were performed for all formulations using a USP II apparatus (paddles) at 100 rpm, with 900 mL of 1.2 pH, 0.1 N HCl, and a 6.8 pH phosphate buffer as dissolution medium. The release of the drug was evaluated by using ultraviolet (UV) spectroscopy.

### 2.18. Formulation with HPMC K100M and Avicel PH102

**Figure 2:**
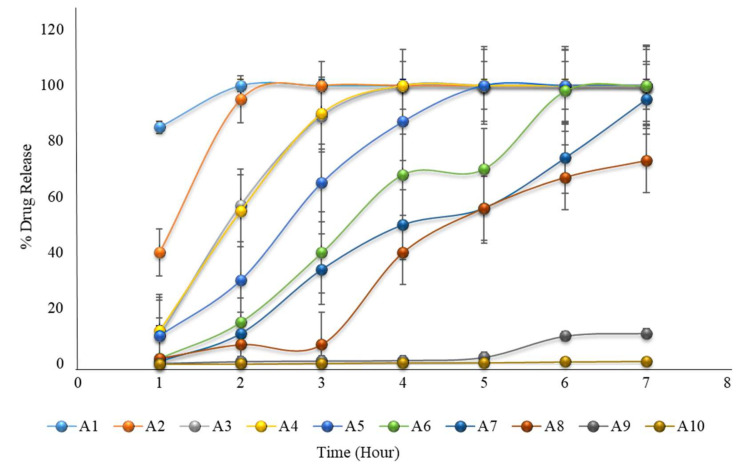
The in vitro dissolution of formulations A1 to A10 (the first 2 h in an acidic medium of pH 1.2, and then in a phosphate buffer medium of pH 6.8) showing that none of the formulations met the expected criteria for pulsatile drug release.

The in vitro dissolution of formulations A1 to A10 is given in Table 5, and a graphical representation is shown in Figure 2. The results showed that none of the formulations met the expected criteria for pulsatile drug release. Formulations A1 to A4 showed complete drug release before 3 to 4 h, and formulation A5 showed complete drug release before 5 h. In these formulations, the concentrations of HMPC K 100M were 10 to 35%. As the concentration of HPMC K100M increased in the formulation of A6 to A10 from 40 to 100%, drug release was not completed within 6 h. As the concentration on HPMC K100M increased, the drug release decreased. Siepmann and Peppas (2012) also concluded that with an increasing coating weight having HPMC, drug release decreased [14].

### 2.19. Formulation with HPMC K100M and Eudragit L100

**Figure 3:**
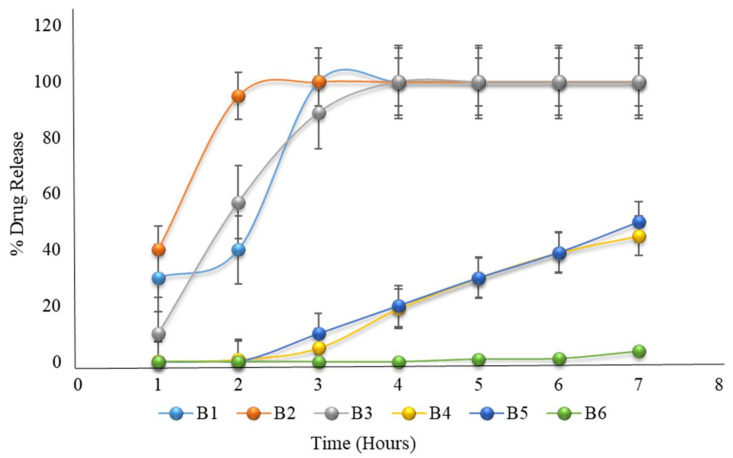
The in vitro dissolution of formulations B1 to B6 (the first 2 h in an acidic medium of pH 1.2, then in a phosphate buffer medium of pH 6.8) mimicked that of the formulations B1 to B3, which had 100% drug release, though formulations B4 to B6 failed to completely release before 6 h.

Formulation B1 to B3 showed a 100% drug release before 3 to 4 h, while formulation B4 to B6 failed to completely release the drug before 6 h; hence, none of the formulations met the criteria for acceptance of pulsatile drug delivery. As discussed for A6 to A10, as the HPMC K100M concentration increased by 40%, the drug release was retarded, while in formulation B4 to B6, the HPMC K100M concentration was above 40%. Eudragit L100 was selected by the authors in their study on a time- and pH-dependent multi-particulate pulsatile drug delivery system [15]. In formulations B1 to B6, Eudragit L100 only contributed to the stability of the system in the acidic environment of the stomach (Table 6 and Figure 3).

### 2.20. Formulation with HPMC K100M and Hydroxyethyl Cellulose

**Figure 4:**
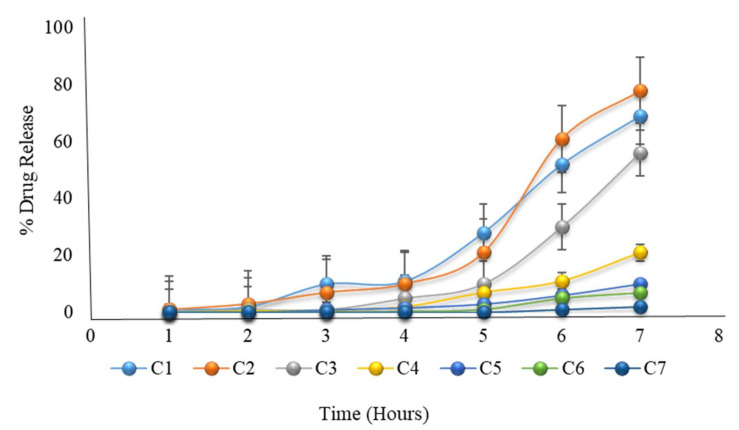
The in vitro dissolution of formulations C1 to C7 (the first 2 h in an acid medium of pH 1.2, and then in a phosphate buffer medium of pH 6.8) showing that all formulations failed to release the drug even after 6 h.

As seen in Table 7 and Figure 4, the results showed that all formulations of the HPMC K100M and HEC combination failed to completely release the drug even after 6 h. As the concentration of HEC increased, drug release decreased because of the high viscosity of HEC. HPMC K100M and HEC are both high viscosity polymers, hence their combination retarded drug release, as previously reported in the literature [16]. 

### 2.21. Formulation with HPMC K100M and HPMC E5 with Constant Coating Weight

**Figure 5:**
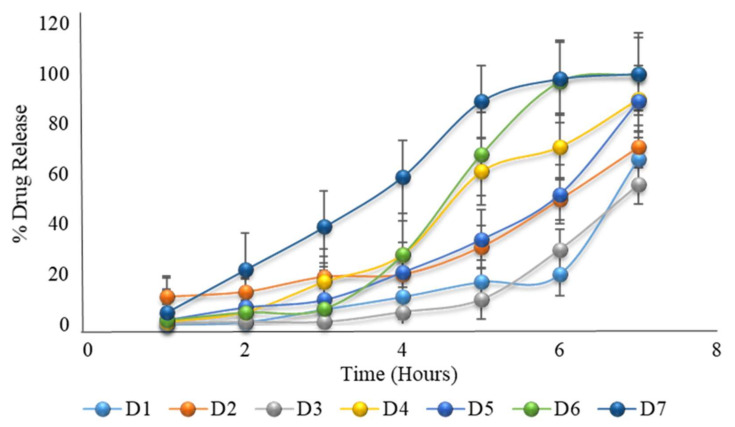
The in vitro dissolution of formulation D1 to D7 (the first 2 h in an acid medium of pH 1.2, and then in a phosphate buffer medium of pH 6.8) showing the desired drug release after a delay time of 5 to 6 h.

As HPMC K100M is a very viscous polymer, when combined with the low viscosity polymer HPMC E5 at a ratio of 12.5% to 87.5%, the desired drug release after five to six hours lag time is achieved (as shown in Table 8 and Figure 5). The authors also developed a pulsatile drug delivery system using HPMC polymers of various viscosity grades [17]. Formulation D6 had very close results, which were expected, but it was not optimized. Formulation D6 was further formulated by changing the coating weight and analyzing its drug release.

### 2.22. Formulation with HPMC K100M and HPMC E5 with Variable Coating Weight

**Figure 6:**
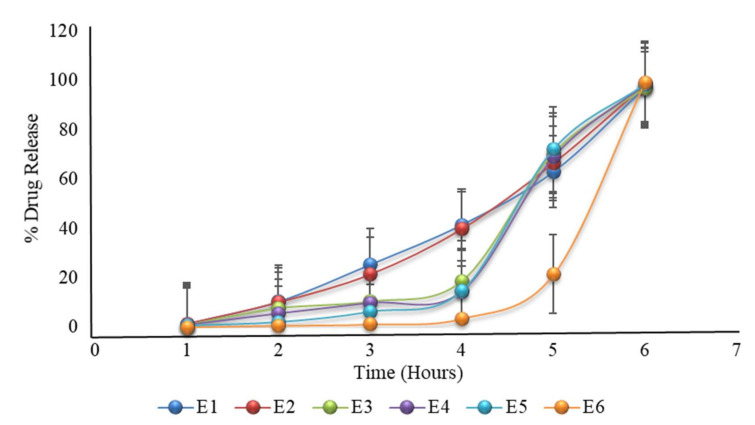
The in vitro dissolution of formulation E1 to E6 (the first 2 h in an acidic medium of pH 1.2, then in a phosphate buffer medium of pH 6.8) prepared after modifying the formulation of D6. The E6 formulation showed promising drug release, as required.

Modifying the formulation of D6, having a varying coated weight, also showed variation in drug release. E6 showed 3.5%, 21.4%, and 99.27% drug release after four hours, five hours, and six hours, respectively (Table 9 and Figure 6); hence formulation E6 was the optimized formulation. The results showed that with increasing the coating weight, there was a decrease in drug release. The same criteria were also reported in the literature, where lag time and coat weight had a direct relation, as with the increase in lag time, coating weight ultimately increased [18].

### 2.23. Stability Studies

As the results showed, formulation E6 was the optimized formulation. This formulation was subjected to stability studies and the results of the percentage of drug release after 1, 2, and 3 months are tabulated in Table 10, and in Table 11 the assay of the selected formulation is tabulated. The results of the stability studies showed that the formulation was stable under different temperature and humidity conditions, as mentioned in the methodology.

## 3. Methodology

### 3.1. Materials

Aceclofenac was obtained from Highnoon Laboratories Ltd. (Lahore, Pakistan); Avicel PH102 was obtained from JRS Pharma (Rosenberg, Germany); croscarmellose sodium was obtained from Mingtai (Taoyuan City, Taiwan ); magnesium stearate was obtained from Peter Greven Asia (Selangor, Malaysia); sunset yellow lake E110 was obtained from Roha, A JJT Group Company (Nagpur, India); HPMC K100M and HPMC E5 were obtained from Zhongbao Chemicals Co., Ltd (Hangzhou, China); Eudragit L100 was obtained from Evonik (Essen, Germany); and hydroxyethyl cellulose was obtained from Sigma Aldrich (St. Louis, MO, USA). All excipients were of standard pharmacopoeia grade and all chemical reagents used for analysis were of analytical grade.

HPMC is available in a variety of viscosity grades. Viscosities of the aqueous solution of methocel are measured at 20 °C. The viscosity of HPMC K100M is 100 cp, while the viscosity of HPMC E5 is 5 cp. Hydroxyethyl cellulose is soluble in water, is a non-ionic polymer, and, in a 2% solution, has a viscosity of 80–125 cp at 20 °C. Eudragit L100 is a delayed release polymer that dissolves at 6.0 pH, and croscarmellose sodium (Ac-di-sol) is a cross-linking polymer derived from carboxymethylcellulose sodium. Ac-di-sol is used as a disintegrant. Microcrystalline cellulose is partially depolarized, purified cellulose that is available as a white, tasteless, odorless, crystalline powder having porous particles. Microcrystalline cellulose is available in different particle sizes and moisture grades having different applications and properties. Avicel PH102 has a particle size of 100 µm.

HPMC K100M, HPMC E5, and HEC were selected for the coatings because they have the tendency to swell, form gel, and erode when they come in contact with water. With such properties, they play a role in delaying the release of the inner core. Eudragit L100 is a pH-dependent polymer and only resists drug release in the acidic media of the stomach.

### 3.2. Methods

In this study, tablet-in-table technology was used for the preparation of press-coated tablets of varying rations [19].

### 3.3. Preparation of the Core Tablets

All the core tablets were formulated by the direct compression method. Three formulations, F1, F2, and F3, were prepared with variable concentrations of croscarmellose sodium, as mentioned in Table 12. An accurately weighed quantity of aceclofenac, microcrystalline cellulose, and croscarmellose sodium were sifted through mesh number 40 (a). The color was mixed with a small quantity of microcrystalline cellulose, further mixed geometrically with microcrystalline cellulose, and then sifted through mesh number 40 (b). After both siftings, the sieved materials of steps (a) and (b) were added to the double cone mixer and mixed thoroughly for 10 min to ensure uniformity. The accurately weighed magnesium stearate was first sifted through mesh number 40 and then added to the uniform blend for 3 min to ensure proper mixing. The tablets were prepared using a 9 mm round concave punch on a ZP 19 rotary press [3].

### 3.4. Preparation of the Press-Coated Tablets

After evaluation of the core tablets, the selected formulation was compress-coated with different blends of polymers. All the different combinations used to prepare the press-coated tablets are mentioned in Table 13 (HPMC K100M and Avicel PH102) as formulation A1 to A10, Table 14 (HPMC K100M, Eudragit L100, and Avicel PH102) as formulation B1 to B6, Table 15 (HPMC K100M and hydroxyethyl cellulose) as formulation C1 to C7, and Table 16 (HPMC K100M and HPMC E5) as formulation D1 to D7, while the formulation of D6 was modified in terms of coating weight and six more formulations (E1 to E6) were formulated, with the quantities mentioned in Table 17.

For the coating blend, the polymers and other excipients were sifted through mesh number 40 and blended in a double cone mixer for ten minutes. Magnesium stearate was then added, and the mixture was further blended for 3 min. The press-coated tablets were manufactured by using a single punch tableting machine. The core tablet was placed into the middle of a powder bed weighing exactly half of the polymer, having been added into the die. Then the remaining half quantity of the polymer mixture was added to the die, and the contents were compressed under a compression pressure of 2 tons using a round concave punch with a 12 mm diameter [20].

### 3.5. Evaluation of the Powder Blend

The powder blend was characterized according to the following parameters, as mentioned in the literature.

#### 3.5.1. Micromeritic Properties

By evaluating micromeritics, the nature of powder blend can be depicted as flow properties and a compressibility tendency for tablet formulation [21]. The pre-compression powder was characterized by its micromeritic characteristics, for example, angle of repose, bulk density, tapped density, and compressibility index.

#### 3.5.2. Angle of Repose (θ)

The angle of repose is a test that measures inter-particulate friction, or the resistance to movement between particles. For very fine and sticky materials, the angle of repose is high. Materials with a low angle of repose are highly flowable and can be transported using gravitational force or little energy. The angle of repose was characterized by the greatest angle possible between the outside of the powder heap and the flat plane. A funnel was fixed with its tip at a given height (*h*) over a level flat surface on which graph paper was placed. The mixed powder was carefully poured until it filled the funnel and the peak of the cone-shaped heap just contacted the tip of the funnel [22]. The angle of repose was measured by the formula given in Equation (1):

(1) $$Ɵ=tan^{-1\;}\;\frac{h}{r}$$

where “θ” is the angle of repose, “*h*” is the height of the heap of the powder blend, and “*r*” is the radius of the heap of the powder blend.

#### 3.5.3. Bulk Density

This test is used to determine the amount of powder that can fit into a space, such as a blender or a hopper on a tablet press. It is the proportion of the weight of the powder blend to the mass volume. Bulk density depends upon the particle size distribution, shape, and cohesiveness of the particles. An amount of accurate weight of powder mixture was carefully filled into a graduated measuring cylinder through a large funnel, and the volume was calculated. This is referred to as initial bulk volume [18,23]. The unit of bulk density is gm/cc and is calculated by using the formula given in Equation (2):

(2) $$D_{b}=\frac{M}{V_{0}}$$

where D_b_ is the bulk density (gm/cc), *M* is the mass of the powder blend (g), and V_0_ is the bulk volume of the powder blend (cc).

#### 3.5.4. Tapped Density (D_t_)

Tapped density is a pharmacopeial test. It is determined by the ratio of the mass of the powder and the volume taken by the powder after tapping the cylinder for a specified time. For calculating D_t_, 10 gm of powder mixture was poured into a 100 mL measuring cylinder. This cylinder was tapped 100 times from a fixed height, and the tapped volume of the powder blend was measured [23]. The tapped density is expressed as gm/cc, and is calculated by using the formula given in Equation (3):

(3) $$D_{t}=\frac{M}{V_{t}}$$

where Dt is the tapped density (gm/cc), M is the mass of the powder blend (gm), and V_t_ is the tapped volume of powder blend (cc).

#### 3.5.5. Carr’s Compressibility Index

The compressibility index of the mixed powder was calculated using the Carr compressibility index.

The Carr’s index was determined by using the formula given in Equation (4):

(4) $$\mathrm{Carr}’s\;\mathrm{Index}\;(\%)=\frac{\mathrm{Tapped}\;\mathrm{Density}\;-\;\mathrm{Bulk}\;\mathrm{Density}}{\mathrm{Tapped}\;\mathrm{Density}}\;\times \;100$$

### 3.6. Evaluation of Post-Compression Parameters

All the prepared tablets were characterized according to the following parameters, as mentioned in the literature.

#### 3.6.1. Hardness Test

A hardness test was used to determine the structural integrity of the tablets. A total of 10 tablets were picked randomly from each formulation, and their hardness was checked by using a Pharmatron MultiTest 50 hardness tester [24]. The values of the mean and standard deviation were calculated.

#### 3.6.2. Thickness

A thickness test was used to find out the thickness of the tablets. For calculating thickness, 10 tablets were picked randomly from each formulation, and each tablet was checked using digital vernier calipers [24]. The mean and standard deviation values were calculated.

#### 3.6.3. Friability Test

A friability test was used to determine how much mechanical stress the tablets were able to withstand during their manufacture, distribution, and handling by the patients. The friability test apparatus was utilized to test the friability of the tablets. Initially, ten tablets (W_1_) were weighed and added to the friabilator. The friabilator was turned on for one-hundred revolutions. The tablets were then weighed again (W_2_) [11]. The percentage of friability was calculated by using the formula given in Equation (5):

(5) $$F=\frac{W_{1}-W_{2}}{W_{1}}\;\times \;100$$

#### 3.6.4. Weight Variation Test

A weight variation test was performed to determine the individual weight variation of the tablets using an average weight in order to determine the consistency of uniformity of the formulations. Twenty tablets were randomly selected from each formulation and the weight of each individual tablet was determined. The average weight was calculated, from which the percentage of deviation was calculated (Equation (6)). Tablets passed the test if not more than two tablets fell outside the percentage limit and none of the tablets differed by more than double the percentage limit [11].

(6) $$\mathrm{Percentage}\;\mathrm{Deviation}=\frac{(W_{\mathrm{average}})-\left(W_{\mathrm{initial}}\right)}{\left(W_{\mathrm{average}}\right)}\;\times \;100$$

#### 3.6.5. Disintegration Time

This test was used to calculate the time it took for the tablets to disintegrate. The disintegration test apparatus was used to determine the in vitro disintegration time of all the prepared tablets of different formulations. The core tablets were placed in each of the six tubes of the apparatus.

#### 3.6.6. Assay of Core Tablets

An assay of the core tablets was performed after taking 20 tablets, weighing them, and grinding them in a mortar. A quantity of powder exactly equivalent to 100 mg of aceclofenac was taken and added to a 100 mL volumetric flask. Approximately 60 mL of methanol was added, and the flask was treated in an ultrasonic bath for 10 min until complete disintegration was achieved. The mixture was allowed to cool to an ambient temperature and the volume was made up with methanol, then stirred magnetically for 15 min. Approximately 5 mL of this solution was transferred to a 50 mL volumetric flask, and the volume was made up with a mobile phase. The absorbance area was measured using HPLC. The test conditions were standard volume and the sample injection was 20 µL. The column used was a Purospher STAR RP-18 150 × 4.6 mm (5 µm). The flow rate was 1.5 mL/min. The temperature was set at 250 °C and the rotation time was about 10 min. The detector used was UV at a wavelength of 276 nm [25].

#### 3.6.7. Drug Content Uniformity

Ten tablets were taken in 10 different 100 mL volumetric flasks. Approximately 75 mL of methanol was added to each flask and then vigorously shaken for 30 min using an ultrasonic bath. The mixtures were cooled to room temperature and diluted to volume with methanol, then filtered through Whatman filter paper. The first 20 mL of filtrate was discarded and the mixture was further diluted by 5 mL to 50 mL with a mobile phase. The absorbance area was measured using HPLC. The test conditions were standard volume and the sample injection was 20 µL. The column used was a Purospher STAR RP-18 150 × 4.6 mm (5 µm). The flow rate was 1.5 mL/min. The temperature was set at 30 °C and the rotation time was about 10 min. The detector used was UV at a wavelength of 276 nm [26].

#### 3.6.8. In Vitro Dissolution Study

The in vitro dissolution study was performed using the USP II (paddle) dissolution test apparatus [27]. Dissolution of the press-coated tablets was carried out using a phosphate buffer of pH 6.8, except for formulations B1 to B6, where the first two hours of dissolution was carried out in acidic medium having 0.1 N HCl and then in a phosphate buffer medium of pH 6.8. The volume of the media was 900 mL, the rotation speed was 100 rpm, and the temperature was maintained at 37 °C ± 0.5 °C. After every 1 h, 5 mL of sample was withdrawn and replaced with the same volume of dissolution media. The sample was filtered through a 0.45 µm disc filter and then further diluted by 1 mL to 10 mL with dissolution media. The standard solution prepared by accurately weighing 10 mg of aceclofenac in a 100 mL volumetric flask, dissolving, and then making it up to 100 mL volume with methanol. Approximately 10 mL of this resulting solution was transferred into a 100 mL volumetric flask and volume was added with dissolution medium. The absorbance of the standard and test solutions was measured at 276 nm using a UV spectrophotometer. The contents of aceclofenac were calculated using the formula given in Equation (7):

(7) $$\%\;\mathrm{age}=\frac{A_{T}\;\times W_{S}}{A_{S\;}\times \;\mathrm{LC}}\;\times \;0.9\;\times \;P\;\times \;100$$

where A_T_ is the absorbance of aceclofenac in the sample solution, A_S_ is the absorbance of aceclofenac in the standard solution, W_S_ is the weight of aceclofenac, WR (mg) of P is the purity of aceclofenac (as a percentage), and LC is the labeled claim per tablet (100 mg).

#### 3.6.9. Stability Studies

In this research work, the stability studies of the selected formulation (E6) were performed according to the International Council for Harmonization (ICH) guidelines at 25 °C ± 2 °C/60% ± 5% RH, 30 °C ± 2 °C/65% ± 5% RH, and 40 °C ± 2 °C/75% ± 5% RH for 3 months. After a specific time interval, parameters such as physical appearance, drug contents, and dissolution were evaluated [28,29].

## 4. Conclusions

In the present research project, press-coated tablets of aceclofenac were prepared by using HPMC K100M, HPMC E5, and HEC. From this study, it can be concluded that the press-coated tablets of aceclofenac containing HPMC K100M and HPMC E5 in a ratio of 12.5 to 87.5% at a 600 mg compression weight (formulation E6) showed negligible drug release after 4 h and maximum drug release after 5 and 6 h, which was the objective of the pulsatile drug delivery system. Formulation E6 was considered optimum. Stability studies of the E6 formulation showed that the product was stable throughout the study period (three months).

## Figures and Tables

**Table 1:** The characterization of aceclofenac.

Description	Specification	Observation
Appearance	A white or off-white crystalline powder	Off-white crystalline powder
Identification	FTIR	Complies
Appearance of solution	Not more turbid than standard	Complies
Loss on drying	≤0.5%	0.2%
Melting point	149–151 °C	151 °C
Assay	99.0–101.0%	100.1%

**Table 2:** The pre-compression tests of the core tablets (F1 to F3).

Formulation	F1	F2	F3
Bulk Density (gm/cm^3^)	0.55 ± 0.015	0.56 ± 0.02	0.56 ± 0.005
Tapped Density (gm/cm^3^)	0.60 ± 0.017	0.62 ± 0.05	0.61 ± 0.009
Angle of Repose (θ)	27 ± 0.01	27 ± 0.20	28 ± 0.02
Carr’s Index (%)	8.3 ± 0.012	9.67 ± 0.04	8.19 ± 0.05
Hausner’s Ratio	1.09 ± 0.02	1.11 ± 0.02	1.10 ± 0.03

**Table 3:** Characterization of the post-compression core tablets (F1 to F3).

Formulation	F1	F2	F3
Diameter (mm)	9.01 ± 0.01	9.00 ± 0.01	9.01 ± 0.01
Thickness (mm)	2.91 ± 0.02	2.96 ± 0.01	2.92 ± 0.011
Hardness (kp)	5.3 ± 0.17	5.56 ± 0.05	5.53 ± 0.05
Average weight (mg)	150.15 ± 1.53	150.1 ± 1.41	150.15 ± 0.81
Friability (%)	0.5 ± 0.01	0.2 ± 0.01	0.2 ± 0.01
Assay (%)	99.92 ± 0.04	99.94 ± 0.13	99.98 ± 0.01
Content uniformity (%)	99.87 ± 0.06	101.25 ± 0.07	99.97 ± 0.02
Dissolution (%)	98.91 ± 0.15	98.9 ± 0.14	99.95 ± 0.03

**Table 4:** Physical characteristics of the press-coated formulations.

Formulation	Thickness (mm)	Hardness (kp)	Average Weight (mg)	Friability (%)
A1	4.38 ± 0.05	13.4 ± 0.02	502 ± 0.5	0.6 ± 0.01
A2	4.42 ± 0.07	11.8 ± 0.03	499 ± 1.1	0.6 ± 0.01
A3	4.41 ± 0.08	13.1 ± 0.06	500 ± 0.7	0.5 ± 0.02
A4	4.50 ± 0.06	11.4 ± 0.04	501 ± 1.0	0.7 ± 0.01
A5	4.25 ± 0.11	10.1 ± 0.03	501 ± 0.4	0.4 ± 0.03
A6	4.10 ± 0.07	12.5 ± 0.01	498 ± 0.07	0.4 ± 0.02
A7	4.27 ± 0.04	12.0 ± 0.05	499 ± 0.13	0.6 ± 0.01
A8	4.27 ± 0.05	13.7 ± 0.04	502 ± 0.6	0.6 ± 0.01
A9	4.34 ± 0.07	13.2 ± 0.04	501 ± 0.09	0.6 ± 0.01
A10	4.55 ± 0.04	13.5 ± 0.07	503 ± 0.5	0.4 ± 0.01
B1	4.41 ± 0.02	12.1 ± 0.06	499 ± 0.42	0.5 ± 0.01
B2	4.11 ± 0.04	12.3 ± 0.05	499 ± 0.33	0.4 ± 0.02
B3	4.47 ± 0.03	11.7 ± 0.04	501 ± 0.09	0.5 ± 0.01
B4	4.44 ± 0.03	11.5 ± 0.02	502 ± 0.11	0.7 ± 0.01
B5	4.52 ± 0.02	11.7 ± 0.04	501 ± 0.09	0.6 ± 0.01
B6	5.45 ± 0.01	12.0 ± 0.03	498 ± 0.11	0.5 ± 0.03
C1	4.34 ± 0.01	12.4 ± 0.08	501 ± 0.13	0.5 ± 0.02
C2	4.38 ± 0.04	13.0 ± 0.12	498 ± 1.2	0.4 ± 0.03
C3	4.42 ± 0.02	13.5 ± 0.04	500 ± 0.8	0.3 ± 0.02
C4	4.40 ± 0.05	12.7 ± 0.06	502 ± 0.17	0.6 ± 0.01
C5	4.51 ± 0.08	11.4 ± 0.06	502 ± 1.1	0.7 ± 0.01
C6	4.49 ± 0.04	12.2 ± 0.05	499 ± 0.7	0.6 ± 0.01
C7	4.33 ± 0.07	12.5 ± 0.08	497 ± 0.14	0.5 ± 0.02
D1	4.41 ± 0.06	12.3 ± 0.08	498 ± 0.14	0.5 ± 0.02
D2	4.5 ± 0.02	12.4 ± 0.10	501 ± 0.11	0.4 ± 0.03
D3	4.4 ± 0.02	13.4 ± 0.06	499 ± 0.09	0.6 ± 0.01
D4	4.38 ± 0.05	11.7 ± 0.09	502 ± 0.17	0.5 ± 0.01
D5	4.42 ± 0.01	11.9 ± 0.05	501 ± 0.14	0.6 ± 0.01
D6	4.53 ± 0.04	12.5 ± 0.06	501 ± 0.09	0.5 ± 0.02
D7	4.37 ± 0.02	12.4 ± 0.09	502 ± 1.1	0.4 ± 0.01
E1	4.29 ± 0.02	11.7 ± 0.06	473 ± 0.14	0.7 ± 0.01
E2	4.42 ± 0.04	12.4 ± 0.11	501 ± 0.23	0.6 ± 0.01
E3	4.59 ± 0.01	12.8 ± 0.09	524 ± 0.18	0.0.7 ± 0.01
E4	4.77 ± 0.01	13.1 ± 0.08	552 ± 0.09	0.5 ± 0.02
E5	4.87 ± 0.03	13.7 ± 0.06	574 ± 0.11	0.5 ± 0.02
E6	5.08 ± 0.03	14 ± 0.06	602 ± 0.14	0.5 ± 0.01

**Table 5:** The in vitro dissolution of formulations A1 to A10 (the first 2 h in an acidic medium of pH 1.2, then in a phosphate buffer medium of pH 6.8).

Time	Percent Drug Release (%)									
Hour	A1	A2	A3	A4	A5	A6	A7	A8	A9	A10
1	85	40	10	12	10	2	1	2	0.2	0.01
2	100	95	57	55	30	15	11	7	0.9	0.04
3	100	100	89	90	65	40	34	7	1.1	0.24
4	100	100	100	100	87	68	50	40	1.3	0.5
5	100	100	100	100	100	70	56	56	2.4	0.51
6	100	100	100	100	100	98	74	67	10	0.8
7	100	100	100	100	100	100	95	73	11	1

**Table 6:** The in vitro dissolution of formulations B1 to B6 (the first 2 h in an acidic medium of pH 1.2, then in a phosphate buffer medium of pH 6.8).

Time	Percent Drug Release (%)					
Hour	B1	B2	B3	B4	B5	B6
1	30	40	10	0.2	0	0
2	40	95	57	0.9	0	0
3	100	100	89	5	10	0
4	100	100	100	19	20	0
5	100	100	100	30	30	1
6	100	100	100	39	39	1.2
7	100	100	100	45	50	4

**Table 7:** The in vitro dissolution of formulations C1 to C7 (the first 2 h in an acid medium of pH 1.2, and then in a phosphate buffer medium of pH 6.8).

Time	Percent Drug Release (%)						
Hour	C1	C2	C3	C4	C5	C6	C7
1	1	1	0.2	0.1	0.01	0	0
2	2	3	1	0.5	0.1	0	0
3	10	7	1	0.7	1	0	0
4	11	10	5	2	1.7	0.2	0
5	28	21	10	7	3	0.9	0
6	52	61	30	11	6	5	1
7	69	78	56	21	10	7	2

**Table 8:** The in vitro dissolution of formulations D1 to D7 (the first 2 h in an acid medium of pH 1.2, and then in a phosphate buffer medium of pH 6.8).

Time	Percent Drug Release (%)						
Hour	D1	D2	D3	D4	D5	D6	D7
1	0.02	11	0.2	1	2	2	5
2	0.9	13	1	5	7	5	22
3	6	19	1	17	10	6.4	39
4	11	20	5	28	21	28	59
5	17	31	10	61	34	68	89
6	20	50	30	71	52	97	98
7	66	71	56	90	89	100	100

**Table 9:** The in vitro dissolution of formulation E1 to E6 (the first 2 h in an acidic medium of pH 1.2, then in a phosphate buffer medium of pH 6.8).

Time	Percent Drug Release (%)					
Hour	E1	E2	E3	E4	E5	E6
1	1.42	1.4	1	0.9	0.75	0.08
2	10.4	10.1	7.7	5.5	2.2	0.71
3	25.4	21.4	10.4	9.8	6.4	1.2
4	41.5	39.8	18.7	14.49	14.8	3.5
5	63	66.4	70.5	68.9	72.17	21.4
6	97.06	98.01	97.06	98.46	98.45	99.27

**Table 10:** Stability studies of the E6 formulation under different temperatures and humidity conditions (n = 3).

Time (Hours)	Dissolution after 1 Month (% Drug Release)			Dissolution after 2 Months (% Drug Release)			Dissolution after 3 Months (% Drug Release)		
Temp (°C)/ Humidity (%)	25 °C/60%	30 °C/65%	40 °C/75%	25 °C/60%	30 °C/65%	40 °C/75%	25 °C/60%	30 °C/65%	40 °C/75%
1	0.09 *±* 0.01	0.08 *±* 0.04	0.07 *±* 0.01	0.08 *±* 0.01	0.07 *±* 0.01	0.06 *±* 0.04	0.09 *±* 0.02	0.07 *±* 0.03	0.08 *±* 0.01
2	0.69 *±* 0.03	0.67 *±* 0.03	0.71 *±* 0.03	0.68 *±* 0.04	0.65 *±* 0.03	0.68 *±* 0.12	0.70 *±* 0.03	0.67 *±* 0.01	0.65 *±* 0.04
3	1.3 *±* 0.02	1.2 *±* 0.06	1.0 *±* 0.03	1.3 *±* 0.06	1.1 *±* 0.05	1.1 *±* 0.07	1.3 *±* 0.02	1.2 *±* 0.01	1.0 *±* 0.04
4	3.6 *±* 0.02	3.4 *±* 0.01	3.1 *±* 0.07	3.4 *±* 0.04	3.4 *±* 0.03	3.3 *±* 0.04	3.6 *±* 0.07	3.5 *±* 0.02	3.2 *±* 0.02
5	21.1 *±* 0.03	21.0 *±* 0.02	20.8 *±* 0.05	21.5 *±* 0.02	21.2 *±* 0.01	20.4 *±* 0.02	21.5 *±* 0.03	21.2 *±* 0.10	20.7 *±* 0.03
6	99.34 *±* 0.03	99.32 *±* 0.03	99.27 *±* 0.03	99.38 *±* 0.05	99.37 *±* 0.04	99.31 *±* 0.03	99.33 *±* 0.07	99.33 *±* 0.03	99.22 *±* 0.02

**Table 11:** Assay of stability samples (n = 3).

Assay	Storage Conditions		
	25 ± 2 °C/60% ± 5% RH	30 ± 2 °C/65% ± 5% RH	40 ± 2 °C/75% ± 5% RH
Initial	99.98% *±* 0.01	99.98% *±* 0.04	99.98% *±* 0.02
After 1st month	99.94% *±* 0.03	99.92% *±* 0.06	99.92% *±* 0.05
After 2nd month	99.90% *±* 0.03	99.90% *±* 0.05	99.89% *±* 0.03
After 3rd month	99.89% *±* 0.03	99.83% *±* 0.03	99.82% *±* 0.08

**Table 12:** The formulations of the core tablets (F1, F2, and F3).

Tablet Ingredient *	F1	F2	F3
Aceclofenac	100	100	100
Croscarmellose sodium	0.75	1.5	3.75
Avicel PH102	47.6	46.85	44.6
Magnesium stearate	1.5	1.5	1.5
Sunset yellow lake E100	0.15	0.15	0.15
Total weight of tablets	150	150	150

* The quantities of all the ingredients are in mg.

**Table 13:** The formulations of A1 to A10.

Formulation *	HPMC K100M	Avicel PH102
A1 (10%)	35	311.5
A2 (20%)	70	276.5
A3 (25%)	87.5	259
A4 (30%)	105	241.5
A5 (35%)	122.5	224
A6 (40%)	140	206.5
A7 (45%)	157.5	189
A8 (50%)	175	171.5
A9 (60%)	210	136.5
A10 (99%)	346.5	0

* Each formulation had 3.5 mg of magnesium stearate. The weight of the core tablet was 150 mg, and the total weight of the press-coated tablet was 500 mg. In all the formulations, the quantities of HPMC K100M and Avicel PH102 were in mg.

**Table 14:** The formulations of B1 to B6.

Formulation * % (HPMC K100M:Eudragit L100)	HPMC K100M (mg)	Eudragit L100 (mg)	Avicel PH102 (mg)
B1 (10:30)	35	105	206.5
B2 (20:20)	70	70	206.5
B3 (30:10)	105	35	206.5
B4 (40:10)	140	35	171.5
B5 (45:15)	157.5	52.5	136.5
B6 (50:20)	175	70	101.5

* Each formulation had 3.5 mg of magnesium stearate. The weight of the core tablet was 150 mg, and the total weight of the press-coated tablet was 500 mg.

**Table 15:** The formulations of C1 to C7.

Formulation * % (HPMC K100M:HEC)	HPMC K100M (mg)	HEC (mg)
C1 (100:0)	346.5	0
C2 (87.5:12.5)	303.19	43.31
C3 (75:25)	260.74	85.76
C4 (50:50)	173.25	173.25
C5 (25:75)	85.76	260.74
C6 (12.5:87.5)	43.31	303.19
C7 (0:100)	0	346.5

* Each formulation had 3.5 mg of magnesium stearate. The weight of the core tablet was 150 mg, and the total weight of the press-coated tablet was 500 mg.

**Table 16:** The formulations of D1 to D7.

Formulation *	HPMC K100M	HPMC E5
D1 (100:0)	346.5	0
D2 (87.5:12.5)	303.19	43.31
D3 (75:25)	260.74	85.76
D4 (50:50)	173.25	173.25
D5 (25:75)	85.76	260.74
D6 (12.5:87.5)	43.31	303.19
D7 (0:100)	0	346.5

* Each formulation had 3.5 mg of magnesium stearate. The weight of the core tablet was 150 mg, and the total weight of the press-coated tablet was 500 mg. In all the formulations, the quantities of both polymers were in mg.

**Table 17:** The formulations of E1 to E6.

Formulation * HPMC K100M: HPMC E5 (12.5:87.5)	HPMC K100M (mg)	HPMC E5 (mg)	Magnesium Stearate (mg)	Press-Coated Tablet Weight (mg)
E1	40.22	281.53	3.25	475
E2	43.31	303.19	3.5	500
E3	46.406	324.844	3.75	525
E4	49.5	346.5	4	550
E5	52.59	368.16	4.25	575
E6	55.69	389.81	4.5	600

* Each formulation had a core tablet weight of 150 mg.

## Data Availability

Data is contained within the article.

## References

[1] Moon A., Kondawar M., Shah R. (2011). Formulation and evaluation of press-coated indomethacin tablets for pulsatile drug delivery system. J. Pharm. Res..

[2] Satani R., Chotaliya M., Raval M., Sheth N. (2014). Review on recent trends in press-coated pulse drug delivery system. Int. Bull. Drug Res..

[3] Prasanth V., Mitesh P., Sam T., Abin A. (2012). Formulation and evaluation of enteric coated time release press coated tablets of theophylline for chronopharmacotherapy. Pharm. Lett..

[4] Arslan S.A., Tirnaksiz F. (2010). A nonsteroidal antiinflammatory drug: Aceclofenac. FABA J. Pharm. Sci..

[5] Bushra R., Shoaib M.H., Naeem M.I., Aslam N. (2013). Aceclofenac: A new effective and safe NSAID. Int. J. Drug Deliv. Technol..

[6] Raza K., Kumar M., Kumar P., Malik R., Sharma G., Kaur M., Katare O. (2014). Topical delivery of aceclofenac: Challenges and promises of novel drug delivery systems. BioMed Res. Int..

[7] The British Pharmacopoeia Commission Secretariat of the Medicines and Healthcare Products Regulatory Agency (MHRA) (2015). British Pharmacopoeia.

[8] Sackey J., Olowosulu A.K., Abdulsamad A., Gwary S. (2019). Design and evaluation of time dependent delayed-release diclofenac sodium tablets for chronopharmaceutical drug delivery. Br. J. Pharm..

[9] Abdullah E.C., Geldart D. (1999). The use of bulk density measurements as flowability indicators. Powder Technol..

[10] Geldart D., Abdullah E., Hassanpour A., Nwoke L., Wouters I. (2006). Characterization of powder flowability using measurement of angle of repose. China Particuol..

[11] The British Pharmacopoeia Commission Secretariat of the Medicines and Healthcare Products Regulatory Agency (MHRA) (2016). British Pharmacopoeia.

[12] Solomon Hambisa S.B., Suleman S. (2019). In vitro comparative quality assessment of different brands of norfloxacin tablets available in Jimma, Southwest Ethiopia. Drug Des. Dev. Ther..

[13] Al-anbagi M.S., Rajab N.A., Khalil Y.I. (2018). Preparation and characterization of timed drug delivery system of sumatriptan using natural polymers. Iraqi J. Pharm. Sci..

[14] Siepmann J., Peppas N.A. (2012). Modeling of drug release from delivery systems based on hydroxypropyl methylcellulose (HPMC). Adv. Drug Deliv. Rev..

[15] Chauhan D., Shah S. (2012). Formulation and evaluation of pulsatile drug delivery system of aceclofenac for treatment of rheumatoid arthritis. Int. J. Pharm. Pharm. Sci..

[16] Iffat W., Shoaib M.H., Yousuf R.I., Qazi F., Mahmood Z.A., Muhammad I.N., Ahmed K., Ahmed F.R., Imtiaz M.S. (2020). Use of Eudragit RS PO, HPMC K100M, Ethyl Cellulose, and Their Combination for Controlling Nicorandil Release from the Bilayer Tablets with Atorvastatin as an Immediate-Release Layer. J. Pharm. Innov..

[17] Sabitha G., Punitha S., Saikrishna K., Uppuluru A.K., Anusha J., Swapna R. (2011). Formulation and evaluation of aceclofenac Microbeads-A chronotherapeutic approach. J. Pharm. Res..

[18] Barzegar Jalali M., Siyahi Shadbad M.R., Barzegar-Jalai A., Adibkia K., Mohammadi G., Aghai B., Zeraati M. (2006). Design and evaluation of delayed-release osmotic capsule of acetaminophen. Iran. J. Pharm. Sci..

[19] Mittal S., Sandip K., Jobin J. (2014). Design, development and evaluation of pulsatile drug delivery system using tablet in tablet formulation. World J. Pharm. Sci..

[20] Malpure P.S., Chaudhari P.D., Ajab A.B., Sanap D.A., Bhagat H.D. (2009). Formulation and Evaluation of Enteric Coated Timed-Release Press-Coated Tablets for Colon Targeting. Indian J. Pharm. Educ. Res..

[21] Yasmin R., Shoaib M.H., Ahmed F.R., Qazi F., Ali H., Zafar F. (2020). Aceclofenac fast dispersible tablet formulations: Effect of different concentration levels of Avicel PH102 on the compactional, mechanical and drug release characteristics. PLoS ONE.

[22] Savale S.K. (2016). Formulation and Evalution of Aceclofenac Sustained Released Tablet. World J. Pharm. Pharm. Sci..

[23] Dave V., Yadav S., Sharma S., Vishwakarma P., Ali N. (2015). Novel approach of aceclofenac fast dissolving tablet. Pak. J. Pharm. Sci..

[24] Khlebnikova E. (2012). Statistical tools for process qualification. Process Qualification Demonstrating Process Acceptability Lifecycle Approach to Process.

[25] Cartwright A.C. (2016). The British Pharmacopoeia, 1864 To 2014: Medicines, International Standards and the State.

[26] Bhardwaj S., Jain V., Jat R., Mangal A., Jain S. (2010). Formulation and evaluation of fast dissolving tablet of aceclofenac. Int. J. Drug Deliv..

[27] Harish K.H., Dinesh M., Jayanthi C., Joshi H. (2019). In vitro dissolution studies of some commercial brands of aceclofenac tablets. World J. Pharm. Res..

[28] Kaur M., Kaur G., Kaur H., Sharma S. (2013). Overview on stability studies. Int. J. Pharm. Chem. Biol. Sci..

[29] Ojha A., Bhargava S. (2022). International Council for Harmonisation (ICH) guidelines. Regulatory Affairs in the Pharmaceutical Industry.

